# 4-Nitro-*N*′-[(*E*)-3-pyridylmethyl­idene]benzohydrazide

**DOI:** 10.1107/S1600536810011244

**Published:** 2010-03-31

**Authors:** Tanveer Ahmad, Muhammad Zia-ur-Rehman, Hamid Latif Siddiqui, Shahid Mahmud, Masood Parvez

**Affiliations:** a Institute of Chemistry, University of the Punjab, Lahore 54590, Pakistan; b Applied Chemistry Research Centre, PCSIR Laboratories Complex, Lahore 54600, Pakistan; c Department of Chemistry, The University of Calgary, 2500 University Drive NW, Calgary, Alberta, Canada T2N 1N4

## Abstract

In the title moleclue, C_13_H_10_N_4_O_3_, the methyl­idene–hydrazide [–C(=O)—N—N=C–] fragment is essentially planar, with a maximum deviation of 0.0228 (7) Å. The mean planes of the benzene and pyridine rings make dihedral angles of 25.44 (6) and 5.47 (7)°, respectively, with the mean plane of the methyl­idene–hydrazide fragment. In the crystal structure, inter­molecular N—H⋯N hydrogen bonds link mol­ecules into chains along the *b* axis. Additional stabilization is provided by weak inter­molecular C—H⋯O hydrogen bonds. The O atoms of the nitro group are disordered over two sets of sites of equal occupancy.

## Related literature

For the synthesis of related compounds, see: Zia-ur-Rehman *et al.* (2009 ▶). For the biological activity of benzohydrazides, see: Chakraborty & Patel (1996 ▶). For closely related structures, see: Raj *et al.* (2008 ▶); Fun *et al.* (2008 ▶); Wang *et al.* (2008 ▶); Qiu *et al.* (2009 ▶).
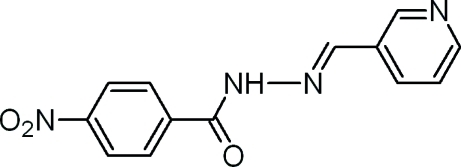


## Experimental

### 

#### Crystal data

C_13_H_10_N_4_O_3_
*M* *_r_* = 270.25
Monoclinic, 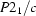

*a* = 14.6158 (3) Å
*b* = 8.1969 (2) Å
*c* = 10.3645 (2) Å
β = 100.609 (1)°
*V* = 1220.49 (5) Å^3^
*Z* = 4
Cu *K*α radiation
μ = 0.91 mm^−1^
*T* = 123 K
0.20 × 0.16 × 0.05 mm

#### Data collection

Bruker APEXII diffractometer
Absorption correction: multi-scan (*SADABS*; Bruker, 2004 ▶) *T*_min_ = 0.839, *T*_max_ = 0.956
10114 measured reflections
2192 independent reflections
2098 reflections with *I* > 2σ(*I*)
*R*_int_ = 0.016

#### Refinement

*R*[*F* ^2^ > 2σ(*F* ^2^)] = 0.036
*wR*(*F* ^2^) = 0.094
*S* = 1.06
2192 reflections
190 parameters
66 restraints
H-atom parameters constrained
Δρ_max_ = 0.26 e Å^−3^
Δρ_min_ = −0.26 e Å^−3^

### 

Data collection: *APEX2* (Bruker, 2004 ▶); cell refinement: *SAINT* (Bruker, 2004 ▶); data reduction: *SAINT* and *XPREP* (Bruker, 2004 ▶); program(s) used to solve structure: *SHELXS97* (Sheldrick, 2008 ▶); program(s) used to refine structure: *SHELXL97* (Sheldrick, 2008 ▶); molecular graphics: *ORTEP-3* (Farrugia, 1997 ▶); software used to prepare material for publication: *SHELXL97*.

## Supplementary Material

Crystal structure: contains datablocks global, I. DOI: 10.1107/S1600536810011244/lh5020sup1.cif

Structure factors: contains datablocks I. DOI: 10.1107/S1600536810011244/lh5020Isup2.hkl

Additional supplementary materials:  crystallographic information; 3D view; checkCIF report

## Figures and Tables

**Table 1:** Hydrogen-bond geometry (Å, °)

*D*—H⋯*A*	*D*—H	H⋯*A*	*D*⋯*A*	*D*—H⋯*A*
N2—H2*N*⋯N4^i^	0.88	2.09	2.9108 (14)	154
C8—H8⋯O3^ii^	0.95	2.50	3.1423 (14)	125
C11—H11⋯O2^iii^	0.95	2.49	3.364 (8)	152
C11—H11⋯O2′^iii^	0.95	2.52	3.371 (8)	150
C13—H13⋯O3^iv^	0.95	2.57	3.1279 (14)	118

Symmetry codes: (i) 
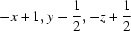
; (ii) 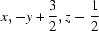; (iii) 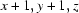; (iv) 
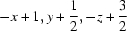
.

**Fig. 1.**
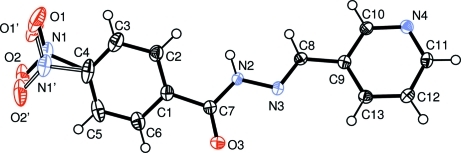
The asymmetric unit of the title compound with the displacement ellipsoids plotted at 50% probability level (Farrugia, 1997); nitro group was disordered over two sites.

## supplementary crystallographic information

### Comment

Schiff bases are well known for their anti-bacterial, anti-oxidant, and anti-tumor activities (Zia-ur-Rehman *et al.*, 2009). These are also considered as popular ligands in coordination chemistry due to their ease of synthesis and their ability to be readily modified both electronically and sterically (Chakraborty & Patel, 1996). We have synthesized a novel Schiff base, by the condensation of pyridine-3-carbaldehyde with *p*-nitrobenzohydrazide, and determined its crystal structure which is presented in this paper.

The structure of the title compound is presented in Fig. 1. The bond distances and angles agree with the cortresponding bond distances and angles reported in closely related compounds (Raj *et al.*, 2008; Fun *et al.*, 2008; Wang *et al.*, 2008; Qiu *et al.*, 2009). The methylidenehydrazide fragment C7/C8/N2/N3/O3 in the title compound is essentially planar with maximum deviation being 0.0228 (7) Å for both C7 and N2 atoms. The mean-planes of the benzene ring (C1–C6) and pyridine ring (C9–C13/N4) make dihedral angles of 25.44 (6) and 5.47 (7)°, respectively, with the mean-plane of the methylidene hydrazide fragment. The structure is stabilized by extensive hydrogen bonding; details have been provided in Table 1.

### Experimental

A mixture of para-nitrobenzohydrazide (0.5 g, 2.76 mmoles), pyridine-3-carbaldehyde (0.26 ml, 2.76 mmoles), orthophosphoric acid (0.2 ml) and methanol (50.0 ml) was heated to reflux for a period of 3.5 hours followed by removal of the solvent under vacuum. The contents were allowed to cool and washed with cold methanol to yield the title compound. Crystals suitable for X-ray crystallographic studies were grown from a methanol solution of the title compound at room temperature by slow evaporation. Yield: 92%. M.p. 547 K.

### Refinement

Though all the H atoms could be distinguished in the difference Fourier map the H-atoms bonded to C-atoms were included at geometrically idealized positions and refined in riding-model approximation with N—H = 0.88 Å and C—H = 0.95 Å; the *U*_iso_(H) were allowed at 1.2*U*_eq_(C/N). The final difference map was essentially featurless. The nitro group was disordered over two sites with N and O atoms occupying equal site occupancy factors, commands SIMU and EADP in SHELXL-97 (Sheldrick, 2008) were used to model the disorder.

### Crystal data

**Table:**

C_13_H_10_N_4_O_3_	*F*(000) = 560
*M**_r_* = 270.25	*D*_x_ = 1.471 Mg m^−^^3^
Monoclinic, *P*2_1_/*c*	Melting point: 547 K
Hall symbol: -P 2ybc	Cu *K*α radiation, λ = 1.54178 Å
*a* = 14.6158 (3) Å	Cell parameters from 10114 reflections
*b* = 8.1969 (2) Å	θ = 3.0–68.0°
*c* = 10.3645 (2) Å	µ = 0.91 mm^−^^1^
β = 100.609 (1)°	*T* = 123 K
*V* = 1220.49 (5) Å^3^	Plate, yellow
*Z* = 4	0.20 × 0.16 × 0.05 mm

### Data collection

**Table:**

Bruker APEXII diffractometer	2192 independent reflections
Radiation source: fine-focus sealed tube	2098 reflections with *I* > 2σ(*I*)
graphite	*R*_int_ = 0.016
ω and φ scans	θ_max_ = 68.0°, θ_min_ = 3.0°
Absorption correction: multi-scan (*SADABS*; Bruker, 2004)	*h* = −17→17
*T*_min_ = 0.839, *T*_max_ = 0.956	*k* = −9→9
10114 measured reflections	*l* = −12→12

### Refinement

**Table:**

Refinement on *F*^2^	Primary atom site location: structure-invariant direct methods
Least-squares matrix: full	Secondary atom site location: difference Fourier map
*R*[*F*^2^ > 2σ(*F*^2^)] = 0.036	Hydrogen site location: difference Fourier map
*wR*(*F*^2^) = 0.094	H-atom parameters constrained
*S* = 1.06	*w* = 1/[σ^2^(*F*_o_^2^) + (0.0485*P*)^2^ + 0.519*P*] where *P* = (*F*_o_^2^ + 2*F*_c_^2^)/3
2192 reflections	(Δ/σ)_max_ = 0.001
190 parameters	Δρ_max_ = 0.26 e Å^−^^3^
66 restraints	Δρ_min_ = −0.26 e Å^−^^3^

### Special details

**Table:**

Geometry. All esds (except the esd in the dihedral angle between two l.s. planes) are estimated using the full covariance matrix. The cell esds are taken into account individually in the estimation of esds in distances, angles and torsion angles; correlations between esds in cell parameters are only used when they are defined by crystal symmetry. An approximate (isotropic) treatment of cell esds is used for estimating esds involving l.s. planes.
Refinement. Refinement of *F*^2^ against ALL reflections. The weighted *R*-factor wR and goodness of fit *S* are based on *F*^2^, conventional *R*-factors *R* are based on *F*, with *F* set to zero for negative *F*^2^. The threshold expression of *F*^2^ > σ(*F*^2^) is used only for calculating *R*-factors(gt) etc. and is not relevant to the choice of reflections for refinement. *R*-factors based on *F*^2^ are statistically about twice as large as those based on *F*, and *R*- factors based on ALL data will be even larger.

### Fractional atomic coordinates and isotropic or equivalent isotropic displacement parameters (Å^2^)

**Table:**

	*x*	*y*	*z*	*U*_iso_*/*U*_eq_	Occ. (<1)
N1	−0.0353 (11)	0.277 (2)	0.3850 (9)	0.0354 (16)	0.50
O1	−0.086 (3)	0.336 (5)	0.295 (3)	0.059 (3)	0.50
O2	−0.0552 (6)	0.1587 (11)	0.4450 (5)	0.0550 (12)	0.50
N1'	−0.0292 (11)	0.259 (2)	0.4237 (9)	0.0354 (16)	0.50
O1'	−0.082 (3)	0.311 (5)	0.321 (3)	0.059 (3)	0.50
O2'	−0.0525 (6)	0.1572 (11)	0.4997 (5)	0.0550 (12)	0.50
O3	0.36776 (6)	0.53420 (11)	0.67517 (8)	0.0267 (2)	
N2	0.37145 (6)	0.60961 (12)	0.46461 (9)	0.0179 (2)	
H2N	0.3442	0.6031	0.3817	0.022*	
N3	0.45517 (6)	0.69107 (12)	0.49965 (9)	0.0183 (2)	
N4	0.66815 (7)	1.02086 (12)	0.31200 (10)	0.0203 (2)	
C1	0.23641 (8)	0.46958 (15)	0.51171 (12)	0.0203 (3)	
C2	0.17661 (9)	0.52595 (17)	0.40038 (13)	0.0261 (3)	
H2	0.1965	0.6096	0.3484	0.031*	
C3	0.08809 (9)	0.46038 (19)	0.36515 (14)	0.0329 (3)	
H3	0.0466	0.4988	0.2898	0.040*	
C4	0.06168 (9)	0.33806 (18)	0.44215 (16)	0.0352 (4)	
C5	0.11925 (10)	0.27997 (17)	0.55309 (16)	0.0361 (4)	
H5	0.0992	0.1953	0.6040	0.043*	
C6	0.20687 (9)	0.34797 (16)	0.58836 (14)	0.0280 (3)	
H6	0.2472	0.3114	0.6655	0.034*	
C7	0.33183 (8)	0.54003 (14)	0.55923 (11)	0.0189 (3)	
C8	0.48376 (8)	0.75796 (14)	0.40288 (11)	0.0182 (3)	
H8	0.4490	0.7430	0.3168	0.022*	
C9	0.56840 (8)	0.85680 (14)	0.42110 (11)	0.0179 (3)	
C10	0.59341 (8)	0.92553 (15)	0.30967 (11)	0.0191 (3)	
H10	0.5552	0.9035	0.2270	0.023*	
C11	0.72083 (8)	1.05187 (15)	0.42965 (12)	0.0212 (3)	
H11	0.7744	1.1186	0.4334	0.025*	
C12	0.70067 (8)	0.99095 (15)	0.54626 (12)	0.0219 (3)	
H12	0.7395	1.0168	0.6277	0.026*	
C13	0.62364 (8)	0.89241 (15)	0.54267 (11)	0.0201 (3)	
H13	0.6085	0.8497	0.6213	0.024*	

### Atomic displacement parameters (Å^2^)

**Table:**

	*U*^11^	*U*^22^	*U*^33^	*U*^12^	*U*^13^	*U*^23^
N1	0.0219 (18)	0.035 (3)	0.051 (5)	−0.0047 (17)	0.010 (4)	−0.013 (4)
O1	0.027 (3)	0.086 (9)	0.059 (8)	−0.015 (4)	−0.004 (5)	−0.022 (6)
O2	0.0311 (8)	0.0435 (8)	0.093 (4)	−0.0147 (6)	0.018 (3)	0.007 (3)
N1'	0.0219 (18)	0.035 (3)	0.051 (5)	−0.0047 (17)	0.010 (4)	−0.013 (4)
O1'	0.027 (3)	0.086 (9)	0.059 (8)	−0.015 (4)	−0.004 (5)	−0.022 (6)
O2'	0.0311 (8)	0.0435 (8)	0.093 (4)	−0.0147 (6)	0.018 (3)	0.007 (3)
O3	0.0277 (5)	0.0329 (5)	0.0186 (4)	−0.0062 (4)	0.0022 (3)	0.0030 (4)
N2	0.0158 (5)	0.0208 (5)	0.0168 (5)	−0.0024 (4)	0.0021 (4)	−0.0006 (4)
N3	0.0156 (5)	0.0187 (5)	0.0205 (5)	−0.0013 (4)	0.0030 (4)	−0.0014 (4)
N4	0.0184 (5)	0.0215 (5)	0.0215 (5)	0.0005 (4)	0.0053 (4)	0.0010 (4)
C1	0.0192 (6)	0.0194 (6)	0.0240 (6)	−0.0001 (4)	0.0080 (5)	−0.0052 (5)
C2	0.0207 (6)	0.0318 (7)	0.0263 (6)	−0.0023 (5)	0.0058 (5)	−0.0034 (5)
C3	0.0207 (6)	0.0429 (8)	0.0345 (7)	−0.0011 (6)	0.0032 (5)	−0.0121 (6)
C4	0.0184 (6)	0.0315 (7)	0.0579 (9)	−0.0057 (5)	0.0130 (6)	−0.0211 (7)
C5	0.0293 (7)	0.0217 (7)	0.0631 (10)	−0.0037 (5)	0.0234 (7)	−0.0006 (7)
C6	0.0252 (6)	0.0227 (6)	0.0387 (7)	0.0003 (5)	0.0126 (5)	0.0015 (6)
C7	0.0197 (6)	0.0167 (6)	0.0206 (6)	0.0005 (4)	0.0045 (4)	−0.0013 (5)
C8	0.0178 (5)	0.0181 (6)	0.0182 (5)	0.0010 (4)	0.0021 (4)	−0.0005 (4)
C9	0.0170 (6)	0.0163 (6)	0.0206 (6)	0.0023 (4)	0.0040 (4)	−0.0012 (4)
C10	0.0180 (6)	0.0196 (6)	0.0193 (6)	0.0012 (4)	0.0024 (4)	−0.0013 (5)
C11	0.0161 (5)	0.0207 (6)	0.0271 (6)	−0.0016 (4)	0.0045 (5)	−0.0013 (5)
C12	0.0198 (6)	0.0235 (6)	0.0212 (6)	−0.0003 (5)	0.0008 (5)	−0.0029 (5)
C13	0.0208 (6)	0.0210 (6)	0.0191 (6)	0.0016 (5)	0.0050 (5)	0.0004 (5)

### Geometric parameters (Å, °)

**Table:**

N1—O1	1.19 (3)	C2—H2	0.9500
N1—O2	1.213 (16)	C3—C4	1.380 (2)
N1—C4	1.518 (16)	C3—H3	0.9500
N1'—O2'	1.236 (15)	C4—C5	1.379 (2)
N1'—O1'	1.27 (2)	C5—C6	1.383 (2)
N1'—C4	1.460 (17)	C5—H5	0.9500
O3—C7	1.2205 (15)	C6—H6	0.9500
N2—C7	1.3532 (15)	C8—C9	1.4617 (16)
N2—N3	1.3826 (13)	C8—H8	0.9500
N2—H2N	0.8800	C9—C10	1.3929 (16)
N3—C8	1.2788 (15)	C9—C13	1.3964 (16)
N4—C10	1.3397 (16)	C10—H10	0.9500
N4—C11	1.3411 (16)	C11—C12	1.3886 (17)
C1—C6	1.3919 (18)	C11—H11	0.9500
C1—C2	1.3920 (18)	C12—C13	1.3806 (17)
C1—C7	1.5061 (16)	C12—H12	0.9500
C2—C3	1.3865 (18)	C13—H13	0.9500
			
O1—N1—O2	124 (2)	C4—C5—H5	120.8
O1—N1—C4	125 (2)	C6—C5—H5	120.8
O2—N1—C4	111.0 (8)	C5—C6—C1	120.48 (13)
O2'—N1'—O1'	125 (2)	C5—C6—H6	119.8
O2'—N1'—C4	124.5 (9)	C1—C6—H6	119.8
O1'—N1'—C4	111 (2)	O3—C7—N2	124.48 (11)
C7—N2—N3	119.29 (9)	O3—C7—C1	120.77 (11)
C7—N2—H2N	120.4	N2—C7—C1	114.75 (10)
N3—N2—H2N	120.4	N3—C8—C9	121.82 (10)
C8—N3—N2	113.72 (9)	N3—C8—H8	119.1
C10—N4—C11	117.17 (10)	C9—C8—H8	119.1
C6—C1—C2	119.84 (12)	C10—C9—C13	117.86 (11)
C6—C1—C7	116.99 (11)	C10—C9—C8	117.52 (10)
C2—C1—C7	123.10 (11)	C13—C9—C8	124.57 (11)
C3—C2—C1	120.18 (13)	N4—C10—C9	123.95 (11)
C3—C2—H2	119.9	N4—C10—H10	118.0
C1—C2—H2	119.9	C9—C10—H10	118.0
C4—C3—C2	118.43 (14)	N4—C11—C12	123.02 (11)
C4—C3—H3	120.8	N4—C11—H11	118.5
C2—C3—H3	120.8	C12—C11—H11	118.5
C5—C4—C3	122.72 (12)	C13—C12—C11	119.31 (11)
C5—C4—N1'	110.9 (4)	C13—C12—H12	120.3
C3—C4—N1'	126.3 (4)	C11—C12—H12	120.3
C5—C4—N1	126.4 (4)	C12—C13—C9	118.67 (11)
C3—C4—N1	110.8 (4)	C12—C13—H13	120.7
C4—C5—C6	118.33 (13)	C9—C13—H13	120.7
			
C7—N2—N3—C8	−177.02 (10)	N1—C4—C5—C6	178.1 (7)
C6—C1—C2—C3	−0.52 (19)	C4—C5—C6—C1	−1.3 (2)
C7—C1—C2—C3	−177.23 (11)	C2—C1—C6—C5	1.50 (19)
C1—C2—C3—C4	−0.57 (19)	C7—C1—C6—C5	178.40 (11)
C2—C3—C4—C5	0.7 (2)	N3—N2—C7—O3	−5.01 (17)
C2—C3—C4—N1'	177.6 (8)	N3—N2—C7—C1	174.08 (9)
C2—C3—C4—N1	−177.4 (6)	C6—C1—C7—O3	−23.95 (17)
O2'—N1'—C4—C5	2.3 (16)	C2—C1—C7—O3	152.85 (12)
O1'—N1'—C4—C5	−180 (2)	C6—C1—C7—N2	156.92 (11)
O2'—N1'—C4—C3	−174.9 (10)	C2—C1—C7—N2	−26.28 (17)
O1'—N1'—C4—C3	3(3)	N2—N3—C8—C9	176.74 (10)
O2'—N1'—C4—N1	168 (6)	N3—C8—C9—C10	−179.54 (11)
O1'—N1'—C4—N1	−14 (4)	N3—C8—C9—C13	−2.28 (18)
O1—N1—C4—C5	174 (3)	C11—N4—C10—C9	−0.60 (17)
O2—N1—C4—C5	−4.7 (15)	C13—C9—C10—N4	1.38 (18)
O1—N1—C4—C3	−8(3)	C8—C9—C10—N4	178.82 (10)
O2—N1—C4—C3	173.3 (9)	C10—N4—C11—C12	−0.44 (17)
O1—N1—C4—N1'	158 (7)	N4—C11—C12—C13	0.65 (18)
O2—N1—C4—N1'	−21 (4)	C11—C12—C13—C9	0.17 (18)
C3—C4—C5—C6	0.2 (2)	C10—C9—C13—C12	−1.11 (17)
N1'—C4—C5—C6	−177.1 (7)	C8—C9—C13—C12	−178.35 (11)

### Hydrogen-bond geometry (Å, °)

**Table:**

*D*—H···*A*	*D*—H	H···*A*	*D*···*A*	*D*—H···*A*
N2—H2N···N4^i^	0.88	2.09	2.9108 (14)	154
C3—H3···O1	0.95	2.37	2.72 (4)	101
C5—H5···O2'	0.95	2.30	2.666 (8)	102
C8—H8···O3^ii^	0.95	2.50	3.1423 (14)	125
C11—H11···O2^iii^	0.95	2.49	3.364 (8)	152
C11—H11···O2'^iii^	0.95	2.52	3.371 (8)	150
C13—H13···O3^iv^	0.95	2.57	3.1279 (14)	118

Symmetry codes: (i) −*x*+1, *y*−1/2, −*z*+1/2; (ii) *x*, −*y*+3/2, *z*−1/2; (iii) *x*+1, *y*+1, *z*; (iv) −*x*+1, *y*+1/2, −*z*+3/2.
